# Acute diverticulitis management: evolving trends among Italian surgeons. A survey of the Italian Society of Colorectal Surgery (SICCR).

**DOI:** 10.1007/s13304-024-01927-y

**Published:** 2024-07-23

**Authors:** Renato Costi, Antonio Amato, Alfredo Annicchiarico, Filippo Montali, TabooSurvey Group, Alfredo Annicchiarico, Adolfo Petrina, Agostino Fernicola, Alba Oliva, Alberto Gerundo, Alberto Porcu, Alberto Stocco, Alberto Vannelli, Aldo Rocca, Alessandro Bergna, Alessandro Coppola, Alessandro Izzo, Alessandro Soave, Alessandro Vitali, Alessia Fassari, Alessio Giordano, Alessio Impagnatiello, Alessio Rollo, Alex Bruno Bellocchia, Alfonso Amendola, Alfredo Savelli, Amedeo Altamura, Amedeo Antonelli, Andrea Balla, Andrea Barberis, Andrea Bottari, Andrea Favara, Andrea Gianmario Di Santo Albini, Andrea Grego, Andrea Guida, Andrea Lauretta, Andrea Lovece, Andrea Marco Tamburini, Andrea Morini, Andrea Pierre Luzzi, Andrea Romboli, Andrea Tufo, Angelo Alessandro Marra, Anna D’Amore, Anna Guariniello, Annadomenica Cichella, Annalisa Comandatore, Annalisa Pascariello, Antonella Usai, Antonia Lavinia Zuliani, Antonino Spinelli, Antonio Bocchino, Antonio Castaldi, Antonio De Leonardis, Antonio Langone, Arcangelo Picciariello, Arianna Petrungaro, Beatrice Torre, Brunella M. Pirozzi, Bruno Nardo, Bruno Scotto, Bruno Sensi, Carini Stefano, Carlo Alberto Manzo, Carlo Galdino Riva, Carlo Gazia, Carlo Giove, Carlo Salvemini, Carmen Sorrentino, Carolina Bartolini, Carolina Castro Ruiz, Gianmaria Casoni Pataccini, Caterina Baldi, Caterina Lastraioli, Caterina Puccioni, Cecilia Bertarelli, Chiara Caricato, Chiara Piceni, Cinzia Tanda, Claudia Armellin, Claudio Guerci, Corrado Bottini, Cosimo Alex Leo, Cristina Bombardini, Cristina De Padua, Cristina Larotonda, Cristina Soddu, Cristine Brooke Pathirannehalage Don, Dajana Glavas, Damiano Caputo, Daniele Fusario, Daniele Massaro, Daniele Morezzi, Daniele Passannanti, Daniele Sambucci, Daniele Zigiotto, Danilo Vinci, Dario Borreca, Dario D’Antonio, Dario Rosini, Dario Somenzi, Daunia Verdi, David Alessio Merlini, Davide Ferrari, Davide Mascali, Diletta Corallino, Domenico Magagnano, Domenico Rosario Iusco, Domenico Vita, Dorena Caruso, Edoardo Forcignanò, Edoardo Virgilio, Elena Bonati, Eleonora Guaitoli, Elio Francesco Favale, Elisa Bolzoni, Elisa Galasso, Elvira Adinolfi, Emanuela Stratta, Emanuele Caruso, Emanuele Damiano Luca Urso, Emanuele Doria, Emanuele Pontecorvi, Emilio Paolo Emma, Enrico Luzietti, Enrico Pinotti, Erica Monati, Erika Boriani, Ernesto Tartaglia, Ester Marra, Eugenia Rosso, Ezio Lombardo, Fabio Ambrosini, Fabio Carbone, Fabio Crescenti, Fabio Medas, Fabrizio D’Acapito, Federica Chimenti, Federica De Robertis, Federico Cappellacci, Federico Cozzani, Federico Festa, Federico Lovisetto, Federico Maggi, Federico Mazzotti, Filippo D’Agostino, Francesca Ascari, Francesca Di Candido, Francesca Foglio, Francesca Laura Nava, Francesca Mazzarulli, Francesca Meoli, Francesca Paola Tropeano, Francesca Pecchini, Francesca Pegoraro, Francesco Bagolini, Francesco Belia, Francesco Bianco, Francesco Caldaralo, Francesco Casti, Francesco Cobellis, Francesco Colli, Francesco Colombo, Francesco Madeddu, Francesco Maria Romano, Francesco Matarazzo, Francesco Menegon Tasselli, Francesco Pata, Francesco Salvetti, Francesco Serra, Gabriele Bislenghi, Gabriele Luciano Petracca, Gabriella Lionetto, Gaia Colletti, Gennaro Mazzarella, Gennaro Perrone, Giacomo Anedda, Giacomo Carganico, Giacomo Fuschillo, Gian Luca Baiocchi, Gian Luigi Canu, Gianluca Baronio, Gianluca Cassese, Gianluca Fucci, Gianluca Mascianà, Gianluca Pellino, Gianluca Rizzo, Gianluigi Moretto, Gianmario Edoardo Poto, Gianpiero Cione, Giorgio Dalmonte, Giorgio Lisi, Giorgio Rossi, Giovanna Berardi, Giovanna Di Scanno, Giovanna Pavone, Giovanni Battista Damiani, Giovanni Braccini, Giovanni Cestaro, Giovanni Guglielmo Laracca, Giovanni Spiezio, Giovanni Tomasicchio, Giulia Bonfanti, Giulia Cerino, Giulia Maria Francesca Marini, Giulia Turri, Giuliano Barugola, Giuliano Lantone, Giulio Iacob, Giuseppe Candilio, Giuseppe Curro, Giuseppe Frazzetta, Giuseppe Navarra, Giuseppe Palomba, Giuseppe Sica, Giuseppe Trigiante, Gregorio Di Franco, Gregorio Romeo, Guglielmo Clarizia, Guglielmo Giannotti, Guido Mantovani, Guido Sciaudone, Harmony Impellizzeri, Helen Yu, Iacopo Monaci, Ilaria Clementi, Imerio Angriman, Immacolata Iannone, Irnerio Angelo Muttillo, Isabella Ameli, Isabella Pezzoli, Jacopo Guerrini, Jacopo Mercuri, Jacopo Nicolò Marin, Jozel Hila, Laura Fortuna, Laura Olivieri, Leandro Siragusa, Leonardo Solaini, Letizia Santandrea, Lidia Oddis, Ljevin Boglione, Loredana Grezio, Lorenzo Casali, Lorenzo Epis, Lorenzo Gallitiello, Lorenzo Pagliai, Lorenzo Petagna, Lorenzo Ramaci, Lorenzo Tosi, Lorenzo Vona, Luca Amadio, Luca Cestino, Luca Domenico Bonomo, Luca Fabris, Luca Ferrario, Luca Morelli, Luca Perin, Luca Resca, Luca Scaravilli, Lucio Selvaggi, Ludovica Vacca, Ludovico Carbone, Luigi Boccia, Luigi Cayre, Luigi Conti, Luigi Eduardo Conte, Luigi Marano, Maddalena Maria Bignone, Manuela Mastronardi, Marci Pellicciaro, Marco Anania, Marco Angrisani, Marco Beggiato, Marco Calussi, Marco Clementi, Marco D’Ambrosio, Marco Giacometti, Marco Livrini, Marco Materazzo, Marco Montorsi, Marco Pericoli Ridolfini, Marco Realis Luc, Margherita Carbonaro, Maria Carmela Giuffrida, Maria Di Salvo, Maria Francesca Chiappetta, Maria Grazia Sibilla, Marianna Capuano, Mariarita Tarallo, Marina Valente, Mario Giuffrida, Mario Pacilli, Mario Sorrentino, Mario Trompetto, Marta Breda, Marta Mozzin, Marta Spalluto, Marzia Franceschilli, Marzia Tripepi, Massimiliano Caccetta, Massimiliano Mistrangelo, Matelda Bencini, Matteo Capuzzo, Matteo Rossini, Mattia Marinelli, Maurizio Rho, Maurizio Romano, Maurizio Roveroni, Mauro Marzano, Mauro Montuori, Mauro Podda, Mauro Pozzo, Mauro Santarelli, Micaela Piccoli, Michela Campanelli, Michele Cricrì, Michele Manara, Michele Manigrasso, Michelle Vilardo, Miriam Biancu, Nicholas Rizzi, Nick Salimian, Nicola Busi, Nicola Cillara, Nicola Di Bartolomeo, Nicola Tartaglia, Nicoletta Sveva Pipitone Federico, Nicolò De Santis, Noemi Laquatra, Noemi Zorzetti, Nunzio Velotti, Olivia Boccia, Oreste Claudio Buonomo, Pamela Milito, Paola Batistotti, Paolina Saullo, Paolo Massucco, Paolo Pizzini, Pasquale Losurdo, Patrizia Rubini, Peter Marinello, Pierantonio Cardinale, Pierluigi Lobascio, Pierpaolo Sileri, Pietro Anoldo, Pietro Fransvea, Pietro Giorgio Calò, Raffaele De Filippi, Raffaele Lombardi, Renato Meccariello, Renato Pietroletti, Riccardo Magarini, Riccardo Marsengo, Riccardo Nascimbeni, Roberta Longhin, Roberta Tutino, Roberto Cammara, Rocco Aversa, Sabino Capuzzolo, Salvatore Bonarrigo, Salvatore Bracchitta, Salvatore Carrabetta, Sara Cecconi, Sara Gobbi, Sara Ingallinella, Sara Marzorati, Sayali Valiyeva, Sebastiano Grassia, Serafino Marino, Sergio Sforza, Silvia Curcio, Silvia Neri, Silvia Puddu, Silvio Caringi, Simona Badalucco, Simona Grande, Simona Pisicchio, Simone Berardi, Simone Bosi, Simone Gargarella, Sofia Esposito, Stefania Angela Piccioni, Stefania Bettoni, Stefano Barbieri, Stefano Rossi, Stefaon Scabini, Teresa Perra, Tommaso Farolfi, Tommaso Guagni, Tommaso Loderer, Tommaso Stecca, Tommaso Violante, Ugo Elmore, Ugo Grossi, Umberto Cocozza, Valentina Rampulla, Valentina Sbacco, Valentina Zucchini, Vania Silvestri, Vincenza Paola Dinuzzi, Vincenzo Adamo, Vincenzo La Vaccara, Vincenzo Papagni, Vincenzo Schiavone, Vittoria Bellato, Zullo Alessia, Gian Andrea Binda

**Affiliations:** 1 Department of Medicine and Surgery, University of Parma, Parma, Italy; 2 Department of General Surgery, Vaio Hospital, Fidenza, Italy; 3 Department of Coloproctology, Sanremo Hospital, Sanremo, IM Italy; 4 General Surgery, Biomedical Institute, Genoa, Italy

**Keywords:** Acute diverticulitis, Diagnosis, Management, Surgery

## Abstract

Acute diverticulitis (AD) is associated with relevant morbidity/mortality and is increasing worldwide, thus becoming a major issue for national health systems. AD may be challenging, as clinical relevance varies widely, ranging from asymptomatic picture to life-threatening conditions, with continuously evolving diagnostic tools, classifications, and management. A 33-item-questionnaire was administered to residents and surgeons to analyze the actual clinical practice and to verify the real spread of recent recommendations, also by stratifying surgeons by experience. CT-scan remains the mainstay of AD assessment, including cases presenting with recurrent mild episodes or women of child-bearing age. Outpatient management of mild AD is slowly gaining acceptance. A conservative management is preferred in non-severe cases with extradigestive air or small/non-radiologically drainable abscesses. In severe cases, a laparoscopic approach is preferred, with a non-negligible number of surgeons confident in performing emergency complex procedures. Surgeons are seemingly aware of several options during emergency surgery for AD, since the rate of Hartmann procedures does not exceed 50% in most environments and damage control surgery is spreading in life-threatening cases. Quality of life and history of complicated AD are the main indications for delayed colectomy, which is mostly performed avoiding the proximal vessel ligation, mobilizing the splenic flexure and performing a colorectal anastomosis. ICG is spreading to check anastomotic stumps’ vascularization. Differences between the two experience groups were found about the type of investigation to exclude colon cancer (considering the experience only in terms of number of colectomies performed), the size of the peritoneal abscess to be drained, practice of damage control surgery and the attitude towards colovesical fistula.

**Supplementary Information:**

The online version contains supplementary material available at 10.1007/s13304-024-01927-y.

## Introduction

Once considered an elderly condition, diverticular disease and its most frequent complication, acute diverticulitis (AD), are spreading in younger population[1, 2] and increasing worldwide by a 3–9.5% [3–5] annual rate, with relevant morbidity and mortality [3], thus becoming a major issue for national health systems [6, 7].

AD may be a challenging condition, as clinical relevance varies widely, ranging from asymptomatic (or pauci-symptomatic) picture to life-threatening conditions, with continuously evolving diagnostic tools, classifications and proposed management.

Differently from the late seventies, when Hinchey first proposed an intraoperative four-stage classification [8], AD diagnosis and severity assessment is presently performed preoperatively, with a pivotal role played by CT scan, both concerning severity assessment and differential diagnosis with colon cancer, eventually leading to the most appropriate management. Considering AD assessment, several authors [9–13] have tried to improve and translate Hinchey’s intraoperative classification in CT preoperative imaging, also including rarer conditions not listed in 1978 classification, including distant, non-pelvic abscesses [9, 11], stenosis [14], extraluminal air [10, 12] or fistula [13], potentially leading to various approaches, ranging from non-operative management, newly introduced specific procedures and surgical resection. Unfortunately, those classifications resulted as being often more descriptive than useful, and 1978 Hinchey’s one is still the most used classification of AD in clinical practice, although it is hampered by well-known “dark zones”, difficult to classify and to treat.

AD management is continuously evolving, too, both in mild cases as well as in severely affected patients. The improvement of antibiotic-regimens efficacy [15, 16], the development of increasingly effective imaging-guided techniques for mini-invasive, non-surgical drainage [17, 18], and the diffusion of laparoscopic technique also in an emergency setting [19, 20], are reshaping the way AD is approached nowadays, widening management options and multiplying the decision-making moments.

Recent guidelines by international scientific societies [21–24], try to throw some light in such a complex subject and provide recommendations allowing for a flexible management in clinical decision-making. Nevertheless, surveys based on national registry databases[25] show that clinical practice is not evolving as generally recommended, probably also owing to difficult AD multidisciplinary management in an emergency setting, unavailability of latest technology in peripheral hospitals, and an increasing trend towards defensive medicine by general surgeons during their duties.

The present survey is aimed at an appraisal of actual clinical practice in AD diagnosis and management in the early 2020s, in order to verify the real spread of recent recommendations and the progressive abandonment of nowadays unjustified behaviors. This 39-item questionnaire explores gray zones and unconsidered situations that can put the surgeon in difficulty, especially in emergency situations and when experience is still limited. For this reason, as a secondary purpose, the present analysis is aimed at identifying different attitudes associated with clinical experience, by stratifying survey responders in young (residents/within 5 years from residency) and expert surgeons.

## Methods

The survey was carried out between November 2022 and February 2023 by the Colorectal Emergency Section of the Italian Society of Colorectal Surgery (SICCR) and the data were collected using an online questionnaire by Google Forms. This survey, named “Taboos in emergency colorectal surgery—Section: diverticulitis”, aims to explore surgeons’ attitude in diagnosis and management in “borderline" situations, either elective or in emergency, that are still not clearly identified by the currently available guidelines and recommendations. The purpose of the survey was explained to all respondents with a brief introduction and respondents were asked to sign a privacy policy consent on a voluntary basis. Both residents and certified surgeons with various experience in general and colorectal surgery were considered eligible for the survey.

**Fig. 1:**
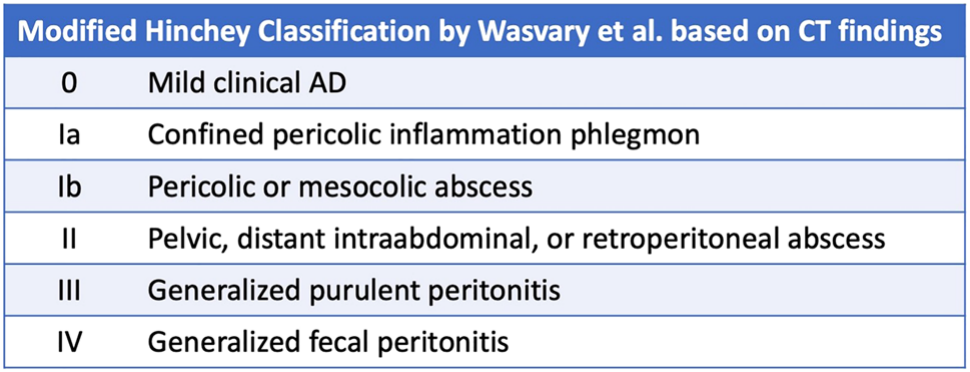
Modified Hinchey classification by Wasvary et al. based on CT findings. *CT* computed tomography, *AD* acute diverticulitis

The Wasvary classification, based on the CT findings and presented at the beginning of the survey was used through all the questionnaire di assess AD severity (Fig. 1).

Responders were invited to answer to a 33-item-questionnaire divided into eight sections: general information, workplace and personal experience in colorectal surgery (Q1–Q10); management of uncomplicated acute diverticulitis (Q11–Q12); management of complicated acute diverticulitis (Q13–Q17); imaging (Q18); elective colectomy (Q19); experience in workplace (Q20–Q21); colovescical fistula (Q22); Surgical technique and technical details of elective colonic resection (Q23–Q33) (Table 1).

**Table 1:** Questions of the survey

General Information, personal experience and workplace workload in colorectal surgery	Q1	Gender
	Q2	Years of experience
	Q3	Workplace activity (colorectal resections per year)
	Q4	Setting of workplace
	Q5	Hospital with emegency (ER, emergency surgery) activities (Y/N)
	Q6	Hospital with intensive care unit (Y/N)
	Q7	Hospital with interventional radiology (Y/N)
	Q8	Workplace region in Italy
	Q9	Number of colectomies performed as operating surgeon
	Q10	Number of laparoscopic procedures performed as operating surgeon
Management—uncomplicated acute diverticulitis	Q11	AD with sigmoid thickening at CT scan (Wasvary Ia), symptomatic stable patient (38 °C hyperpyrexia, elective pain, localized rebound tenderness) in good conditions. Management?
	Q12	What delayed investigation to exclude colon cancer?
Management—complicated acute diverticulitis	Q13	AD with pelvic abscess at CT scan (Wasvary II), stable paucisymptomatic patient (elective pain) in good conditions. From what minimum diameter does the abscess need to be drained?
	Q14	AD with pelvic abscess on CT scan (Wasvary II—pelvic abscess 5 cm in diameter), stable patient with no symptoms in good conditions. The radiologist refuses percutaneous drainage for technical reasons. Management?
	Q15	Symptomatic diffuse peritonitis (38 °C hyperpyrexia, diffuse peritoneal reaction), stable patient in good conditions. CT scan compatible with diffuse purulent peritonitis (Wasvary III). Management?
	Q16	(Referring to the previous clinical situation) At laparoscopic exploration, diffuse purulent peritonitis is confirmed, but a free perforation is not visible. Management?
	Q17	60-year-old patient, hemodynamically stable, symptomatic (hyperpyrexia 38 °C, elective pain, localized rebound tenderness) in good conditions. At CT scan distant pneumoperitoneum (three "bubbles", the largest three centimeters in diameter) with no free fluid. Management?
Imaging	Q18	40-year-old woman with typical symptoms of the umpteenth episode of mild AD (apyretic, elective pain with localized rebound tenderness, slight alteration of inflammation indices). Which test is indicated for diagnostic confirmation?
Delayed elective colectomy	Q19	Indication for delayed, elective colectomy after AD. Which is the main factor?
Experience in your workplace	Q20	In your unit, considering all emergency procedures carried out for AD, which is the percentage of Hartmann's operations?
	Q21	In your environment, in the treatment of patients with hemodynamically unstable AD, is Damage Control Surgery (resection of the sigmoid without anastomosis between the two stumps nor stoma, with temporary closure of the abdomen or laparostomy) an option?
Colo-vescical fistula	Q22	70-year-old patient, active, in good condition, with paucisymptomatic AD-related colo-vesical fistula (mild dysuria, positive urine culture, pneumaturia at CT scan). Management?
Elective colonic resection for diverticular disease of the left colon: surgical technique	Q23	Section of the vessels at the origin (Y/N)
	Q24	Systematic mobilization of the splenic flexure (Y/N)
	Q25	Extension of the resection (sigmoidectomy vs. left colectomy)
	Q26	Proximal margin of the resection (proximal to AD vs. proximal to diverticula)
	Q27	Anastomosis to/below the sacral promontory (Y/N)
	Q28	Anastomosis check: indocyanine green ICG (Y/N)
	Q29	Anastomosis check: hydropneumatic test (Y/N)
	Q30	Anastomosis check: rectoscopy (Y/N)
	Q31	Check of resection rings after mechanical stapling (Y/N)
	Q32	Closure of the mesenteric breach (clips, stitches, glue, other)
	Q33	Perianastomotic drainage (Y/N)

*AD* acute diverticulitis, *Q* question, *Y/N* yes/no

Answering to all questions was mandatory to complete the survey. Survey distribution to surgeons took place through mailing lists, instant message services, and the official social media accounts of the Italian Society of Colorectal Surgery (Società Italiana di Chirurgia Colorettale, SICCR) on Facebook, Instagram, and LinkedIn. A reminder was mailed 2, 4 and 6 weeks after the first mailing. All respondents were informed that the results of the survey would have been used for further statistical evaluation and scientific publication. Anonymity was guaranteed by study design. After the closing date for questionnaire submissions, results were downloaded as a comma separated values (CSV) and analyzed by using Excel (Microsoft Corporation, Redmond, USA). Results of the survey were reported according to the Checklist for Reporting Results of Internet ESurveys (CHERRIES) guidelines [26].

### Statistical analysis

Collected data were processed, and results were summarized as frequencies (*n*) and percentages (%), separately for each question. A further stratification of the outcomes was obtained dividing the responses into two classes of experience according to: number of colectomies performed (≤ 50 vs. ≥ 51) and years of experience (within 5 years from the end of residency program—residents included, vs. > 5 years). All the data were reported in contingency tables for subsequent inferencial analysis. Statistical analyses were performed using the commercial software “SPSS” (IBM SPSS Statistics for Windows [Version 28]. Armonk, NY: IBM Corp.), the open source statistical system “R” (R Core Team (2023). R: A language and environment for statistical computing. R Foundation for Statistical Computing, Vienna, Austria. URL: https://www.R-project.org/), and the freeware package of statistical programs for epidemiologists “Winpepi” (Abramson, J. H. (2016). WinPepi: Computer programs for epidemiologists. [Version 11.65]. Retrieved from http://www.brixtonhealth.com/pepi4windows.html).

The contingency tables obtained from each question were analized using the Chi-squared test and the Fisher’s exact test, as appropriate, to assess the potential difference between experience groups. The results were considered statistically significant for a *p* value below 5% (*p* < 0.05).

## Results

### General information, workplace workload and personal experience in colorectal surgery

**Fig. 2:**
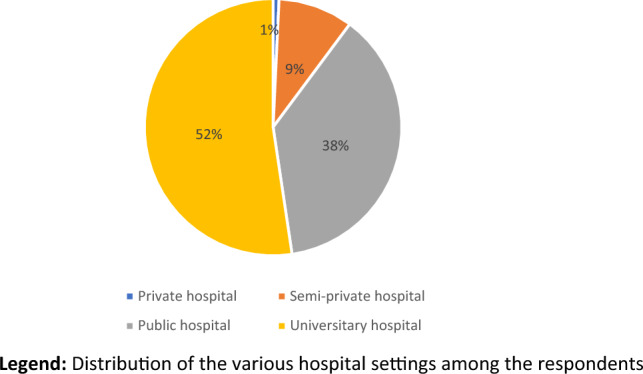
Type of hospital. Distribution of the various hospital settings among the respondents

**Fig. 3:**
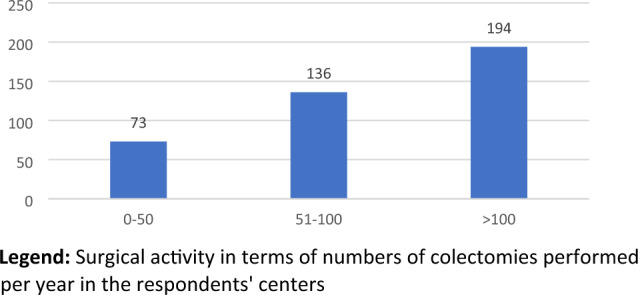
No. of colectomies (unit) per year. Surgical activity in terms of numbers of colectomies performed per year in the respondents' centers

Four hundred and fifty-five questionnaires were correctly completed. After excluding 52 double reports from the same responders, questionnaires from 403 different respondents were considered eligible for the final analysis. Overall, 274 (68%) respondents were men and 128 (31.7%) women (1 was not specified). More than half of the interviewees work in University hospitals (211; 52.3%) (Fig. 2), of these only 73 (18.1%) worked in a hospital where less than 50 colectomies are performed per year (Fig. 3). The majority of expert surgeons (> 5 years of experience) are distributed between university hospitals (61/149; 40.9%) and public hospitals (72/149; 48.3%) while only 10.7% in affiliated private hospitals. The same trend occurs if we consider the respondents by number of colectomies performed. In most environments, an intensive care unit (390; 96.7%) and a regular activity of emergency surgery (354; 87.8%) and interventional radiology (298; 73.9%) were present (Table 2). Table 3 highlights the Italian working regions of the respondents to the survey, compared to SICCR members’ distribution.

**Table 2:** Setting of the hospital

Question	Answer	No.	(%)
Type of hospital	Private hospital	3	0.7
	Semi-private hospital	38	9.4
	Public non-University hospital	151	37.5
	University hospital	211	52.4
No. of colectomies per year	0–50	73	18.1
	51–100	136	33.7
	> 100	194	48.1
Emergency activities	Yes	354	87.8
	No	49	12.2
Interventional radiology unit	Yes	298	73.9
	No	105	26.1
Intensive care unit	Yes	390	96.8
	No	13	3.2

**Table 3:** Working regions of respondents

Region	Survey participants		SICCR members	
	No.	%	No.	%
Abruzzo	12	2.98	24	1.97
Basilicata	1	0.25	2	0.16
Calabria	5	1.24	39	3.20
Campania	42	10.42	93	7.62
Emilia-Romagna	72	17.86	80	6.56
Friuli Venezia Giulia	6	1.49	31	2.54
Lazio	48	11.91	160	13.11
Liguria	16	3.97	51	4.18
Lombardia	69	17	167	13.69
Marche	1	0.25	21	1.72
Molise	3	0.74	5	0.41
Piemonte	22	5.46	123	10.08
Puglia	22	5.46	87	7.13
Sardegna	17	4.22	55	4.51
Sicilia	7	1.74	68	5.57
Toscana	26	6.45	59	4.84
Trentino-Alto Adige	3	0.74	15	1.23
Umbria	1	0.25	26	2.13
Valle d'Aosta	2	0.50	5	0.41
Veneto	28	6.95	109	8.94
Total	403	100	1220	100

**Fig. 4:**
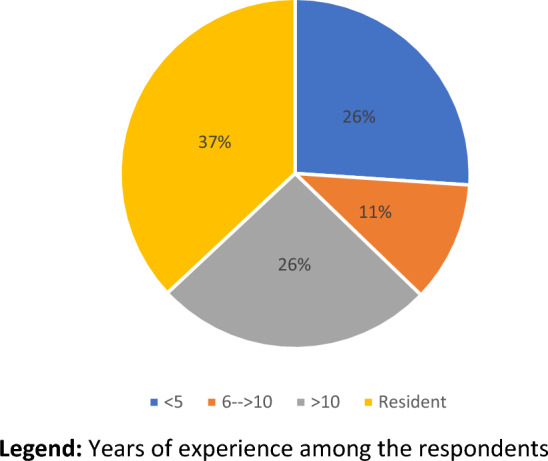
Personal experience (years). Years of experience among the respondents

**Fig. 5:**
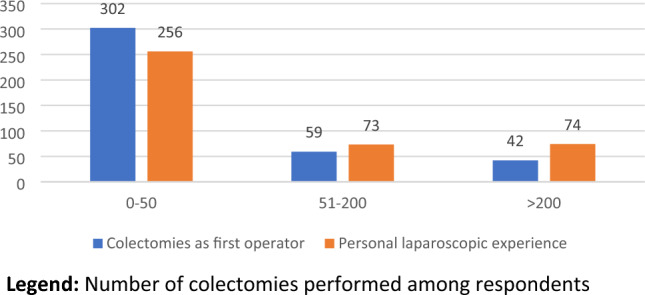
Surgeon’s experience. Number of colectomies performed among respondents

Concerning surgical experience, more than half of interviewed surgeons (254; 63%) were still in their training program or within 5 years from the end of residency, whereas only 104 (25.8%) had more than 10 years of surgical experience (Table 4, Fig. 4). Seventy-five percent (302) had performed fewer than 50 colonic resections in their experience and 18.4% (74) had performed more than 200 laparoscopic procedures (Table 4, Fig. 5).

**Table 4:** Surgeon’s experience

Question	Answer	No.	%
Years of surgical experience	Resident	149	37.0
	< 5 y	105	26.1
	From 6 to 10 y	45	11.2
	> 10 y	104	25.8
Colectomies performed as operating surgeon	0–50	302	74.9
	51–200	59	14.6
	> 200	42	10.4
Laparoscopic procedures performed as operating surgeon	0–50	256	63.5
	51–200	73	18.1
	> 200	74	18.4

### Uncomplicated acute diverticulitis (Table 5)

**Table 5:** Uncomplicated acute diverticulitis (Wasvary Ia) management

Question	Answer	Total (no. 403)		No. of colectomies					Years of experience				
				0–50 (no. 302)		> 50 (no. 101)		*p*	≤ 5 (no. 254)		> 5 (no. 149)		*p*
		No.	%	No.	%	No.	%		No.	%	No.	%	
Question 11	Outpatient. no Ab	13	3.2	8	2.6	5	5.0	0.122	5	2.0	8	5.4	0.074
	Outpatient. oral Ab	167	41.4	129	42.7	38	37.6		112	44.1	55	36.9	
	Hospitalized. IV Ab. no fasting	74	18.4	61	20.2	13	12.9		51	20.1	23	15.4	
	Hospitalized. IV Ab. fasting	147	36.5	103	34.1	44	43.6		86	33.9	61	40.9	
	Other	2	0.5	1	0.3	1	1.0		0	0.0	2	1.3	
Question 12	Virtual colonscopy (or contrast enema enhanced CT) after 60 days	15	3.7	6	2.0	9	8.9	**0.002**	6	2.4	9	6.0	0.111
	Virtual colonoscopy (or contrast enema enhanced CT) after 30 days	24	6.0	14	4.6	10	9.9		12	4.7	12	8.1	
	Colonscopy after 30 days	169	41.9	132	43.7	37	36.6		112	44.1	57	38.3	
	Colonscopy after 60 days	189	46.9	145	48.0	44	43.6		121	47.6	68	45.6	
	Other	6	1.5	5	1.7	1	1.0		3	1.2	3	2.0	

Bold in the tables are statistically significant values

Question 11 = AD with sigmoid thickening at CT scan (Wasvary Ia), symptomatic stable patient (38 °C hyperpyrexia, elective pain, localized rebound tenderness) in good conditions. Management?

Question 12 = What delayed investigation to exclude colon cancer?

*Ab* antibiotics, *IV* intravenous, *d* days

Dealing with the presented case of acute uncomplicated Wasvary Ia diverticulitis (Question 11), 13 (3.2%) surgeons opted for a conservative management without antibiotics, 167 (41.4%) opted for an outpatient management with oral antibiotics, while the remaining ones preferred an intravenous antibiotic therapy as inpatients with (147; 36.5%) or without (74; 18.4%) fasting. Concerning the preferred investigation to be performed to exclude the presence of colon cancer, almost all the interviewees chose colonoscopy, at 30 days (169; 41.9%) or 60 days (189; 46.9%), respectively. Only 39 (9.7%) of surgeons opted for CT with enema (Table 5). The statistical analysis highlighted how the most experienced surgeons (> 50 performed colectomies) express a prefer for enema colon-CT after 30 days (*p* = 0.002).

### Complicated acute diverticulitis (Table 6)

**Table 6:** Complicated acute diverticulitis

Question	Answer	Total (no. 403)		No. of colectomies					Years of experience				
				0–50 (no. 302)		> 50 (no. 101)		*p*	≤ 5 (no. 254)		> 5 (no. 149)		*p*
		No.	%	No.	%	No.	%		No.	%	No.	%	
Question 13	3 cm	32	7.9	27	8.9	5	5.0	**0.0002**	24	9.4	8	5.4	**0.00004**
	4 cm	136	33.7	117	38.7	19	18.8		103	40.6	33	22.1	
	5 cm	155	38.5	107	35.4	48	47.5		84	33.1	71	47.7	
	Diameter is not a factor	70	17.4	43	14.2	27	26.7		35	13.8	35	23.5	
	Other	10	2.5	8	2.6	2	2.0		8	3.1	2	1.3	
Question 14	Transvaginal/transrectal drainage if possible	7	1.7	5	1.7	2	2.0	0.703	6	2.4	1	0.7	0.270
	Laparotomic exploration then evaluation	1	0.2	0	0.0	1	1.0		0	0.0	1	0.7	
	Laparoscopic exploration then W&D	91	22.6	70	23.2	21	20.8		58	22.8	33	22.1	
	Laparoscopic exploration and resection (VL/open. ± ostomy)	23	5.7	15	5.0	8	7.9		11	4.3	12	8.1	
	Wait and see (CT scan at 5–7-day interval)	279	69.2	210	69.5	69	68.3		178	70.1	101	67.8	
	Other	2	0.5	2	0.7	0	0.0		1	0.4	1	0.7	
Question 15	Laparoscopic exploration then evaluation	345	85.6	260	86.1	85	84.2	0.293	219	86.2	126	84.6	0.387
	Laparotomy then evaluation	39	9.7	30	9.9	9	8.9		25	9.8	14	9.4	
	Wait and see (Ab IV. fasting). check every 12 h	17	4.2	10	3.3	7	6.9		8	3.1	9	6.0	
	Other	2	0.5	2	0.7	0	0.0		2	0.8	0	0.0	
Question 16	Laparotomic conversion. Hartmann	30	7.4	21	7.0	9	8.9	0.214	18	7.1	12	8.1	0.149
	Laparotomic conversion. resection and anastomosis with oostomy	33	8.2	25	8.3	8	7.9		19	7.5	14	9.4	
	Laparotomic conversion. resection and anastomosis without oostomy	6	1.5	5	1.7	1	1.0		6	2.4	0	0.0	
	Laparoscopic W&D	179	44.4	131	43.4	48	47.5		105	41.3	74	49.7	
	Laparoscopic Hartmann	39	9.7	34	11.3	5	5.0		29	11.4	10	6.7	
	Laparoscopic resection and anastomosis without oostomy	38	9.4	30	9.9	8	7.9		27	10.6	11	7.4	
	Laparoscopic resection and anastomosis with oostomy	75	18.6	55	18.2	20	19.8		49	19.3	26	17.4	
	Other	3	0.7	1	0.3	2	2.0		1	0.4	2	1.3	
Question 17	Outpatient management. Oral Ab. ambulatorial check in short time	3	0.7	3	1.0	0	0.0	0.169	2	0.8	1	0.7	0.222
	Hospitalization. laparotomic exploration	14	3.5	10	3.3	4	4.0		10	3.9	4	2.7	
	Hospitalization. laparoscopic exploration	112	27.8	91	30.1	21	20.8		77	30.3	35	23.5	
	Hospitalization. IV Ab. short close monitoring	273	67.7	197	65.2	76	75.2		164	64.6	109	73.2	
	Other	1	0.2	1	0.3	0	0.0		1	0.4	0	0.0	

Bold in the tables are statistically significant values

Question 13 = AD with pelvic abscess at CT scan (Wasvary II), stable paucisymptomatic patient (elective pain) in good conditions. From what minimum diameter does the abscess need to be drained?

Question 14 = AD with pelvic abscess on CT scan (Wasvary II—pelvic abscess 5 cm in diameter), stable patient with no symptoms in good conditions. The radiologist refuses percutaneous drainage for technical reasons. Management?

Question 15 = Symptomatic diffuse peritonitis (38 °C hyperpyrexia, diffuse peritoneal reaction), stable patient in good conditions. CT scan compatible with diffuse purulent peritonitis (Wasvary III). Management?

Question 16 = (Referring to the previous clinical situation) At laparoscopic exploration, diffuse purulent peritonitis is confirmed, but a free perforation is not visible. Management?

Question 17 = 60-year-old patient, hemodynamically stable, symptomatic (hyperpyrexia 38 °C, elective pain, localized rebound tenderness) in good conditions. At CT scan distant pneumoperitoneum (three "bubbles", the largest three centimeters in diameter) with no free fluid. Management?

*Ab* antibiotics, *IV* intravenous, *d* days, *W&D* wash and drain

One-hundred-thirty-six (33.7%) of respondents identified 4 cm as the AD-related abscess’ minimum diameter needing to be drained, whereas it was 5 cm for 155 surgeons (38.5%), while it did not influence the decision-making process for 70 (17.4%). The comparative study between less ad more experienced surgeons showed that "5 cm cut-off" is more often preferred by the second ones both in terms of colectomies performed and number of years of experience (*p* < 0.001). When faced with a pelvic abscess non-drainable by radiology techniques, the majority chose a wait-and-see attitude (279; 69.2%), while 91 surgeons (22.6%) would proceed for laparoscopic “lavage and drainage”. Only 7 (1.7%) would opt for vaginal or rectal drainage, and most of these were among the less experienced respondents.

In the presence of radiological and clinical features of purulent diffuse peritonitis (Wasvary III), most of the interviewees (345; 85%) would approach the surgical exploration laparoscopically to confirm CT diagnosis. If a Wasvary III AD is confirmed at laparoscopy, 179 surgeons (44.4%) would perform lavage and drainage only, 75 (18.6%) laparoscopic resection-anastomosis with protective ostomy, and 39 (9.7%) laparoscopic Hartmann resection.

In the case on extraluminal gas (or “air bubbles”), almost all interviewees opted for hospitalization (385; 95.5%), with 112 (27.8%) carrying out a laparoscopic exploration while 273 (67.7%) would prefer a wait and see attitude by antibiotics IV administration and close clinical monitoring (Table 6).

### Imaging (Table 7)

**Table 7:** Imaging

Question	Answer	Total (no. 403)		No. of colectomies					Years of experience				
				0–50 (no. 302)		> 50 (no. 101)		*p*	≤ 5 (no. 254)		> 5 (no. 149)		*p*
		No.	%	No.	%	No.	%		No.	%	No.	%	
Question 18	US by expert radiologist	141	35.0	105	34.8	36	35.6	0.933	90	35.4	51	34.2	0.594
	Clinical exam	26	6.5	19	6.3	7	6.9		14	5.5	12	8.1	
	CT scan	235	58.3	178	58.9	57	56.4		150	59.1	85	57.0	
	Other	1	0.2	0	0.0	1	1.0		0	0.0	1	0.7	

Question 18 = 40-year-old woman with typical symptoms of the umpteenth episode of mild AD (apyretic, elective pain with localized rebound tenderness, slight alteration of inflammation indices). Which test is indicated for diagnostic confirmation?

*US* ultrasounds

Considering the preferred examination to confirm AD etiology in the case of a woman in her childbearing age presenting with the umpteenth episode and mild symptoms, 235 (58.3%) of the interviewees should choose CT scan, 141 (35%) would use ultrasound in expert hands, and 26 (6.5%) would opt for clinical examination alone without carrying out any further investigation (Table 7).

### Elective colectomy (Table 8)

**Table 8:** Main indication for elective colectomy

Question	Answer	Total (no. 403)		No. of colectomies					Years of experience				
				0–50 (no. 302)		> 50 (no. 101)		*p*	≤ 5 (no. 254)		> 5 (no. 149)		*p*
		No.	%	No.	%	No.	%		No.	%	No.	%	
Question 19	Age (≥ o < 50 y)	7	1.7	5	1.7	2	2.0	0.279	4	1.6	3	2.0	0.171
	Impact on QoL	135	33.5	96	31.8	39	38.6		86	33.9	49	32.9	
	No. of previous episodes	99	24.6	81	26.8	18	17.8		69	27.2	30	20.1	
	Immunodeficency	51	12.7	38	12.6	13	12.9		32	12.6	19	12.8	
	Previous abscess treated conservatively	106	26.3	77	25.5	29	28.7		58	22.8	48	32.2	
	Other	5	1.2	5	1.7	0	0.0		5	2.0	0	0.0	

Question 19 = Indication for delayed, elective colectomy after AD. Which is the main factor?

*y* years, *QoL* quality of life, *No.* number

The main indication for delayed, elective colectomy was a significant worsening of perceived quality of life for 135 (33.5%) surgeons, a history of a complicated AD requiring the drainage for 106 (26.3%), whereas for 99 (24.6%) it was the absolute number of previous AD episodes. Fifty-one (12.7%) chose patient’s immuno-deficiency and 7 (1.7%) young age (Table 8).

### Experience in your workplace (Table 9, Fig. 6)

**Table 9:** Unit and personal experience in severe acute diverticulitis

Question	Answer	Total (no. 403)		No. of colectomies					Years of experience				
				0–50 (no. 302)		> 50 (no. 101)		*p*	≤ 5 (no. 254)		> 5 (no. 149)		*p*
		No.	%	No.	%	No.	%		No.	%	No.	%	
Question 20	HP = 25%	117	29.0	93	30.8	24	23.8	0.315	81	31.9	36	24.2	0.107
	HP = 50%	142	35.2	99	32.8	43	42.6		79	31.1	63	42.3	
	HP = 75%	104	25.8	79	26.2	25	24.8		66	26.0	38	25.5	
	HP = > 90%	40	9.9	31	10.3	9	8.9		28	11.0	12	8.1	
Question 21	In my unit we never perform DCS	115	28.5	87	28.8	28	27.7	**0.0014**	77	30.3	38	25.5	**0.000013**
	Yes, I performed DCS as operating surgeon	76	18.9	45	14.9	31	30.7		30	11.8	46	30.9	
	Yes, but I have never performed DCS as operating surgeon	212	52.6	170	56.3	42	41.6		147	57.9	65	43.6	

Bold in the tables are statistically significant values

Question 20 = In your unit, considering all emergency procedures carried out for AD, which is the percentage of Hartmann's operations?

Question 21 = In your environment, in the treatment of patients with hemodynamically unstable AD, is Damage Control Surgery (resection of the sigmoid without anastomosis between the two stumps nor stoma, with temporary closure of the abdomen or laparostomy) an option?

*HP* Hartmann procedure, *DCS* damage control surgery

**Fig. 6:**
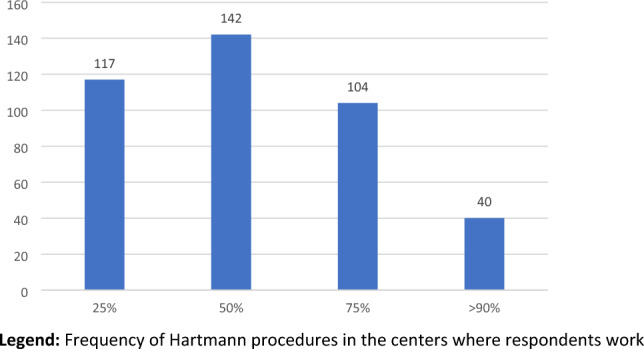
Percentage of Hartmann’s resections/total emergency procedures for AD. Frequency of Hartmann procedures in the centers where respondents work

Asked about the rate of Hartmann operations among all emergency procedures performed for AD at their hospital, 29% of surgeons answered ¼, 35.2% declared ½ and 25.8% reported ¾, with 9.9% of colleagues admitted that such a rate exceeded 90%.

One-hundred-fifteen (28.5%) responders stated that, in their environment, performing damage control surgery (DCS) in a hemodynamically unstable patient is not an option, and 76 (18.9%) declared that they have already performed such a procedure as operating surgeon (Table 9).

The comparison between the two classes of experience, revealed that DCS was more often performed by expert surgeons, both considering colectomies performed (*p* = 0.0014) and years of experience (*p* < 0.001).

### Colovescical fistula (Table 10)

**Table 10:** Acute diverticulitis-related colovescical fistula’s management

Question	Answer	Total (no. 403)	No. of colectomies						Years of experience				
			0–50 (no. 302)			> 50 (no. 101)		*p*	≤5 (no. 254)		> 5 (no. 149)		*p*
		No.	%	No.	%	No.	%		No.	%	No.	%	
Question 22	Resection and anastomosis (no oostomy)	140	34.7	94	31.1	46	45.5	**0.012**	76	29.9	64	43.0	**0.00025**
	Resection and anastomosis (oostomy)	122	30.3	90	29.8	32	31.7		77	30.3	45	30.2	
	Oostomy. imaging in 6 months	48	11.9	41	13.6	7	6.9		39	15.4	9	6.0	
	Endoscopic treatment (± oostomy)	50	12.4	41	13.6	9	8.9		36	14.2	14	9.4	
	Wait and see reevaluation in 6 months	32	7.9	26	8.6	6	5.9		16	6.3	16	10.7	
	Other	11	2.7	10	3.3	1	1.0		10	3.9	1	0.7	

Bold in the tables are statistically significant values

Question 22 = 70-year-old patient, active, in good condition, with paucisymptomatic AD-related colo-vesical fistula (mild dysuria, positive urine culture, pneumaturia at CT scan). Management?

A colovescical fistula in a 70-year-old patient with mild dysuria, positive urine culture, and pneumaturia at CT is reported being mostly approached by colonic resection and anastomosis, without (140, 34.7%) or with (122, 30.3%) protective ostomy. Fifty (12.4%) surgeons would opt for an endoscopic management as first approach (with/without ostomy), 48 (11.9%) would prefer performing just a protective ostomy and re-evaluation at 6 months, while 32 (7.9%) would just wait and re-evaluate the patient at a 6-month-interval (Table 10).

### Surgical technique and technical details (Table 11)

**Table 11:** Surgical technique and details

Q	Answer	Total (no. 403)		No. of colectomies					Years of experience				
				0–50 (no. 302)		> 50 (no. 101)		*p*	≤5 (no. 254)		> 5 (no. 149)		*p*
		No.	%	No.	%	No.	%		No.	%	No.	%	
Q23	Periferal vessels’ section	316	78.4	244	80.8	72	71.3	0.061	203	79.9	113	75.8	0.403
	Vessels’ section at the origin	87	21.6	58	19.2	29	28.7		51	20.1	36	24.2	
Q24	Systematic mobilization of the splenic flexure	235	58.3	169	56.0	66	65.3	0.124	147	57.9	88	59.1	0.898
	Non-systematic mobilization of the splenic flexure	168	41.7	133	44.0	35	34.7		107	42.1	61	40.9	
Q25	Left colectomy	92	22.8	57	18.9	35	34.7	**0.002**	46	18.1	46	30.9	**0.005**
	Sigmoidectomy	311	77.2	245	81.1	66	65.3		208	81.9	103	69.1	
Q26	Above diverticula	212	52.6	167	55.3	45	44.6	0.079	140	55.1	72	48.3	0.224
	Above inflammation	191	47.4	135	44.7	56	55.4		114	44.9	77	51.7	
Q27	Anastomosis proximal to the sacral promontory	85	21.1	69	22.8	16	15.8	0.176	60	23.6	25	16.8	0.134
	Anastomosis at/below the sacral promontory	318	78.9	233	77.2	85	84.2		194	76.4	124	83.2	
Q28	Anastomosis check by indocyanine green	222	55.1	165	54.6	57	56.4	0.842	142	55.9	80	53.7	0.743
	No	181	44.9	137	45.4	44	43.6		112	44.1	69	46.3	
Q29	Anastomosis check by hydropneumatic test	375	93.1	284	94.0	91	90.1	0.262	237	93.3	138	92.6	0.952
	No	28	6.9	18	6.0	10	9.9		17	6.7	11	7.4	
Q30	Anastomosis check by rectoscopy	78	19.4	59	19.5	19	18.8	0.989	52	20.5	26	17.4	0.541
	No	325	80.6	243	80.5	82	81.2		202	79.5	123	82.6	
Q31	Anastomosis check by checking the rings after circular stapling	386	95.8	290	96.0	96	95.0	0.891	243	95.7	143	96.0	0.912
	No	17	4.2	12	4.0	5	5.0		11	4.3	6	4.0	
Q32	Closure of the mesenteric breach by Glue	54	13.4	46	15.2	8	7.9	0.041	38	15.0	16	10.7	0.002
	Closure of the mesenteric breach by Clips	33	8.2	27	8.9	6	5.9		25	9.8	8	5.4	
	Other	127	31.5	99	32.8	28	27.7		90	35.4	37	24.8	
	No	189	46.9	130	43.0	59	58.4		101	39.8	88	59.1	
Q33	Perianastomotic drainage	349	86.6	267	88.4	82	81.2	0.094	225	88.6	124	83.2	0.170
	No	54	13.4	35	11.6	19	18.8		29	11.4	25	16.8	

Bold in the tables are statistically significant values

Question 23 = Section of the vessels at the origin (Y/N)

Question 24 = Systematic mobilization of the splenic flexure (Y/N)

Question 25 = Extension of the resection (sigmoidectomy vs. left colectomy)

Question 26 = Proximal margin of the resection (proximal to AD vs. proximal to diverticula)

Question 27 = Anastomosis to/below the sacral promontory (Y/N)

Question 28 = Anastomosis check: indocyanine green ICG (Y/N)

Question 29 = Anastomosis check: hydropneumatic test (Y/N)

Question 30 = Anastomosis check: rectoscopy (Y/N)

Question 31 = Check of resection rings after mechanical stapling (Y/N)

Question 32 = Closure of the mesenteric breach (clips, stitches, glue, other)

Question 33 = Perianastomotic drainage (Y/N)

*Q* question,* Y/N* yes/no

Concerning elective colectomy technical details, more than ¾ of surgeons opted for a peripheral section of vessels (78.4%), a limited colonic resection (sigmoidectomy, 77.2%) and the colorectal anastomosis at/below the sacral promontory (78.9%). A systematic colonic splenic flexure mobilization was the choice of 58.3% of surgeons.

Among maneuvers aimed at checking colorectal anastomosis integrity, the hydropneumatic test and the check of anastomosis “rings” after mechanical stapling resulted as being performed by 93.1% and 95.8%, respectively, whereas ICG test was reported by 55.1% of surgeons (Table 11).

## Discussion

### Participants and participants’ facilities (Tables 2, 3, 4)

Four-hundred-three general surgeons/general surgery residents responded to the survey, with a 63% rate of young surgeons (within 5 years from residency program). Experience in both major colorectal resections and laparoscopic surgery reported by survey responders are consistent with the young age of most participants. The geographical distribution of participants is related to the numerosity of population in each Italian region as well as SICCR members distribution, with slightly higher rates of responders in Emilia-Romagna, Lombardia and Lazio, probably also owing to survey promotion activity by the authors.

Many responders’ hospitals are secondary–tertiary general hospitals, as only roughly 10% were private health facilities (41/403), only 12.2% without emergency surgery activity (49/403), and 18.1% (73/403) performing less than 50 colorectal resections per year. Interestingly enough, more than ¼ of responders’ hospitals (105/403) does not have interventional radiology unit available on site, which may be supposed to affect AD management, in particular, in the case of complicated cases associated with abscesses (Wasvary II).

The number of University hospitals seems over-represented as they account for more than 50% (211/403), whereas the majority of surgical facilities in Italy are non-University institutions. Such a finding seems related to the high rate of residents (149/403), mostly attending University hospitals, completing the survey.

### AD management

#### Mild AD (Wasvary Ia) (Table 5)

As expected, the vast majority of surgeons favors antibiotic treatment of mild AD cases (Wasvary Ia), whereas it is remarkable that in almost 45% (180) an outpatient setting is adopted. This is consistent with the recent trend in management of non-severe AD cases as outpatients [21, 24]. Interestingly enough, more expert surgeons prefer to associate fasting at inpatients management.

Considering investigations aimed to exclude colorectal cancer after AD episode, colonoscopy (at a 30- or 60-day delay) remains the standard approach for the vast majority of surgeons, regardless of age or experience, while virtual-Colonoscopy (or contrast enema enhanced CT scan), a tool recently proposed by specialized centers for AD assessment [27–29], seems still a niche examination, more frequently preferred by expert colorectal surgeons (*p* = 0.002).

#### Pelvic abscess (Wasvary II) (Table 6)

About ¾ of the participants (291) indicates 4 or 5-cm-diameter the minimum abscess size needed to be drained by imaging-guided-approach. This appears to be consistent with recent literature [24, 30, 31]. The answer "diameter is not a factor", given by as many as 70 surgeons (17.5%), in our opinion should not be recommended, as general and local signs/symptoms may be mild or lack in the case of pelvic abscesses, which therefore could be undertreated. Surprisingly, the rate of this answer is significantly higher in so called expert surgeons (roughly ¼ for both colleagues with > 50 colectomies and > 5-year experience). Probably, such a finding may be supposed to be related to a more updated attitude by less experienced surgeons in such a challenging situation.

### Pelvic abscess management after refused/unsuccessful imaging-guided drainage (Table 6)

In the absence of symptoms or with minor symptoms, a conservative attitude by fluid administration and antibiotic therapy, with reassessment at a 5–7-day interval, is preferred by most surgeons (69%, 279/403). In the case of surgical approach is preferred, a mini-invasive approach of wash and drain (W&D) avoiding major resections in an emergency setting prevails (91/122 undergoing surgery). This conservative and mini-invasive attitude seems to be in line with recent guidelines and, to some extent, confutes the hypothesis of an increasing “defensive” attitude, leading to an early intervention, by surgeons.

#### Purulent peritonitis (Wasvary III) (Table 6)

Surgical exploration is the choice of almost all participants (384/403, 95.3%), with about 10% (39/384) preferring laparotomy to laparoscopy. Considering this latter issue, interestingly enough, laparotomy is preferred by 12% of residents, 8% of young specialists, only 2% of moderately experienced surgeons (5–10 years from residency) and 11.5% among very experienced surgeons. Seemingly, a mid-generation of already experienced, laparoscopy-oriented surgeons also in an emergency setting is progressively substituting the old guard. It is worthwhile, too, that surgeons practicing in environments with less colorectal experience (< 50 colectomies performed yearly) more frequently prefer laparotomy (41%, 30/73) than those working in specialized centers (4%, 9/230).

#### Purulent peritonitis (Wasvary III) without visible perforation (Table 6)

The management of Wasvary III cases without visible perforation divides sourgeons’ opinion, as all answers, ranging from Hartmann operation to unprotected resection-anastomosis, received consideration. Almost one half opts for W&D (179, 44.4%), 152 proceed with resection-anastomosis (unprotected in 119 cases), and 69 prefer a Hartmann procedure, carried out laparoscopically in more than half of cases (39/69). Although with inconclusive results [20, 32], laparoscopic lavage/drainage is a low complexity procedure which has finally entered surgeons’ routine. Although recently discussed [33, 34] W&D is increasingly considered whenever the “hole” is not found at laparoscopy as it may be supposed that in the absence of a communication between colonic lumen and peritoneum, W&D may adequately manage a localized purulent peritonitis. Such a laparoscopy-oriented attitude both performing Hartmann resection and unprotected laparoscopic resection-anastomosis (almost half of the participants overall) in an emergency setting with an ongoing diffuse purulent peritonitis is surprising. Those operations seem complex and technically demanding procedures, potentially associated with significant morbidity, and needing resources not always available in any hospital in an emergency setting. This finding is not consistent with the average experience of survey participants, mostly new specialists or surgeons in training, whose rate of laparoscopic Hartmann procedures is double than that reported by more experienced colleagues. Possibly, personal experience in emergency colorectal surgery and may play a role in preferring an earlier conversion to laparotomy, with shorter operative time.

### Pneumoperitoneum in clinically and hemodynamically stable patients (Table 6)

Traditionally considered a sign of severity and an indication for surgical exploration [10–12], isolated pneumoperitoneum at X-rays/CT-scan has lost its meaning, as evidenced by the wait-and-see attitude of 273 participants (about 2/3), whereas 28% preferred a laparoscopic exploration. Such a conservative attitude, which is more common among expert surgeons than young colleagues, is consistent with recent literature [35, 36].

### Investigation at the umpteenth episode of mild DA in woman of childbearing age (Table 7)

CT is the answer for over 58%, followed by ultrasound in expert hands in 1/3 of the cases (34%). It is possible that the frequent lack of experience in performing AD ultrasound by the radiologist on call in an emergency setting, may eventually push the surgeon to try by objectify AD severity by CT-scan, despite the fertile age should probably lead to a more prudent approach.

### Main indication to delayed elective colectomy (Table 8)

Participants are divided on the main indication to elective colectomy. In accordance with recent trends [22–24, 37], the impact on quality of life (135, 33.5%) and a previous complicated AD episode (106, 26.3%) prevail. Nevertheless, interestingly, the number of surgeons considering surgical resection with respect of the number of episodes, a concept that has been overtaken by literature, remains high (99, 24.6%). It is now almost abandoned, correctly, the (young) age of patients as an indication to sigmoidectomy, which is probably a legacy of the elective aggressive approach proposed during the 80s–90s, even after AD first episode, when the AD recurrence rate was believed to be substantially higher [38]. Significantly, the distribution of answers is not impacted by surgeon’s age and experience.

### Percentage of Hartmann procedures among all emergency operations performed for AD in their environment (Table 9)

According to 259 participants (64%) the number of Hartmann operation is 25–50% of total emergency interventions for AD at their hospital, overall, with no significant differences associated with experience. This is surprising, considering that in US practice, Hartmann procedure is by far the preferred approach (93% out of all emergency operations for AD) [25]. Maybe, geographic differences, possibly associated with a more defensive approach by U.S. surgeons due to a higher impact of medical–legal issues, may explain such a surprising outcome.

### Damage control surgery (colon resection without anastomosis, with/without laparostomy) (Table 9)

Although DCS has been proposed by international guidelines since the early 2010s [39] for the management of AD associated with systemic sepsis/hemodynamic instability, it is somewhat surprising that about 30% of participants does not consider it as an option in the case of severe, life-threatening cases, in their own environment. Not surprisingly, the result is statistically different among young and old surgeons with a percentage of surgeons having performed DCS more than double among esperts (colectomies performed: *p* = 0.0014; years of experience: *p* < 0.001).

### Management of colo-vescical fistula (Table 10)

Colonic resection is the traditional approach to colo-vescical fistula, with or without protection stoma, and is preferred by about 2/3 of participants (262, 65%). A small percentage (50, 12%) consider an endoscopic approach in stable patients as allowed by the latest technological upgrades in fistula endoscopic management [40, 41], which that evidently has not yet entered clinical practice in most environments and remains a niche solution in expert hands. Significantly, a trend towards a higher rate of experienced surgeons preferring unprotected colon resection and lower rates of endoscopy-first attitude, seemingly reflects the impact of experience in such a challenging situation.

### Surgical technique of delayed elective colon resection (Table 11)

According to over ¾ (311, 77.2%) of interviewees, delayed colon resection technique includes after AD includes a limited resection (sigmoidectomy rather than left colectomy), without main vessels (IMV and IMA) section at the origin, with the anastomosis at/under the sacral promontory as suggested by the current recommendations [24]. A systematic mobilization of splenic flexure is performed in almost 60% of cases, while, rather surprisingly, in most cases a colonic section is reported performed “proximally to colonic diverticula". Such ad attitude is not justified, also considering that diverticula often extend far beyond the sigmoid, not rarely reaching the transverse colon. Possibly, participants misunderstood the question and meant "to avoid diverticula" at the point of colon section/anastomosis.

Colorectal anastomosis integrity is almost unanimously checked by verifying the completeness of colon “rings” after circular stapling and by the hydro-pneumatic test. The Indocyanine Green test, a more recently introduced tool to check colonic and rectal stump vascularization at stapling [42, 43], is performed by more than half of participants, showing its recent and progressive spread in surgical practice.

Almost half of surgeons do not close the mesenteric breach in any way (stitches, clips, glue, etc.), which is a viable option according by recent recommendations [44, 45]. Following traditional teaching, roughly 80% of surgeons places a peri-anastomotic drainage, a practice that has recently undergone a critical review, especially if the patient is managed by ERAS (or Fast-Track) protoco [46].

### “Young vs old” and limitations

One of the most ambitious objectives of the survey was to try to provide a differentiation in terms of experience by dividing the responders based on the number of colectomies performed (0–50 vs. > 50) and years of experience (≤ 5 vs. > 5). It is very difficult to define parameters that reflect "experience" and the impact this has on clinical practice. Although this appears to be a stretch and a simplification which may appear excessive given that expertise in colorectal surgery is not a measurable parameter but appears to be a close combination between experience and volume of activity, to our knowledge it seemed like an original effort that could better represent the current snapshot of what really happens in Italian hospitals. Despite expectations, different attitudes between the two experience groups were found only in the type of investigation to exclude colon cancer (difference found only in terms of number of colectomies), the size of the peritoneal abscess to be drained, the use of damage control surgery and in attitudes towards colovesical fistula. A relevant limitation could arise from the fact that the sample taken into consideration comes from the same country and that it is mainly composed of surgeons with little experience. An international survey, which mainly involves more experienced surgeons, and which also has feedback in terms of outcomes, could provide answers with a greater clinical impact. The analysis in the two study groups therefore highlighted minimal differences with very little clinical impact. In this sense, there is an important bias; it would be appropriate to be able to distinguish between responses influenced by real experience and those based exclusively on cultural knowledge of the current guidelines and recommendation and by becoming aware of this it might make more sense to consider the opinion of expert surgeons more.

## Conclusions

The present survey represents an effort to define how AD management is evolving and how recent guidelines and recommendations have spread in surgeons’ practice.

Outpatient management of mild AD is slowly gaining acceptance, whereas small abscesses are largely considered not an indication for drainage until reaching 4–5-cm diameter, as suggested by guidelines.

A conservative management in clinically non-severe cases is spreading, as Wait and see policy is preferred by many in the case of extra-digestive air or non-radiologically drainable abscesses.

In more severe cases (Wasvary III), laparoscopy is largely preferred to laparotomy, as first (and often only) approach. A non-negligible number of surgeons, in particular young ones, seem confident in performing complex procedures in an emergency setting in presence of an ongoing diffuse peritonitis. Such a practice, which by the way is not contraindicated by guidelines, should probably induce some reflection. Surgeons are seemingly aware of several options during emergency surgery for AD, since the rate of Hartmann procedures does not exceed 50% in most environments and damage control surgery is gaining acceptance in the management of life-threatening cases.

CT-scan remains the mainstay of AD assessment, including cases presenting with recurrent mild episodes or women of child-bearing age, where other options should probably be preferred.

The attitude towards delayed, elective colectomy after AD is evolving, with a consistent number of surgeons considering quality of life and history of complicated AD the main indication for resection, consistently with guidelines recommendations.

Delayed elective colectomy for AD is mostly performed in a traditional fashion, avoiding the proximal ligation of main vessels, mostly mobilizing systematically the splenic flexure and performing a low anastomosis at/under the sacral promontory. ICG is spreading as a new tool to check anastomotic stumps’ vascularization.

## Supplementary Information

Below is the link to the electronic supplementary material.

Supplementary file1 (DOCX 11 KB)

Supplementary file2 (DOCX 16 KB)

## Data Availability

Data archiving is not mandated, but data will be made available on reasonable request.

## Declarations

### Statement

The manuscript has not been a podium or poster meeting presentation.
