# Supplementary material for: Acute diverticulitis management: evolving trends among Italian surgeons. A survey of the Italian Society of Colorectal Surgery (SICCR)
Source: Updates Surg. 2024 Jul 23;76(5):1745–60. doi: 10.1007/s13304-024-01927-y (PMC11455713; doi:10.1007/s13304-024-01927-y)
Supplement: Supplementary file 1 — Supplementary file1 (DOCX 11 KB) [file 13304_2024_1927_MOESM1_ESM.docx]

**TabooSurvey Group**

*It is requested that all survey participants be cited on PubMed*

Adolfo Petrina, Agostino Fernicola, Alba Oliva, Alberto Gerundo, Alberto Porcu, Alberto Stocco, Alberto Vannelli, Aldo Rocca, Alessandro Bergna, Alessandro Coppola, Alessandro Izzo, Alessandro Soave, Alessandro Vitali, Alessia Fassari, Alessio Giordano, Alessio Impagnatiello, Alessio Rollo, Alex Bruno Bellocchia, Alfonso Amendola, Alfredo Annicchiarico, Alfredo Savelli, Amedeo Altamura, Amedeo Antonelli, Andrea Balla, Andrea Barberis, Andrea Bottari, Andrea Favara, Andrea Gianmario Di Santo Albini, Andrea Grego, Andrea Guida, Andrea Lauretta, Andrea Lovece, Andrea Marco Tamburini, Andrea Morini, Andrea Pierre Luzzi, Andrea Romboli, Andrea Tufo, Angelo Alessandro Marra, Anna D’Amore, Anna Guariniello, Annadomenica Cichella, Annalisa Comandatore, Annalisa Pascariello, Antonella Usai, Antonia Lavinia Zuliani, Antonino Spinelli, Antonio Bocchino, Antonio Castaldi, Antonio De Leonardis, Antonio Langone, Arcangelo Picciariello

Arianna Petrungaro, Beatrice Torre, Brunella M. Pirozzi, Bruno Nardo, Bruno Scotto, Bruno Sensi, Carini Stefano, Carlo Alberto Manzo, Carlo Galdino Riva, Carlo Gazia, Carlo Giove, Carlo Salvemini, Carmen Sorrentino, Carolina Bartolini, Carolina Castro Ruiz, Casoni Pattacini Gianmaria, Caterina Baldi, Caterina Lastraioli, Caterina Puccioni, Cecilia Bertarelli, Chiara Caricato, Chiara Piceni, Cinzia Tanda, Claudia Armellin, Claudio Guerci, Corrado Bottini, Cosimo Alex Leo, Cristina Bombardini, Cristina De Padua, Cristina Larotonda, Cristina Soddu, Cristine Brooke Pathirannehalage Don, Dajana Glavas, Damiano Caputo, Daniele Fusario, Daniele Massaro, Daniele Morezzi, Daniele Passannanti, Daniele Sambucci, Daniele Zigiotto, Danilo Vinci, Dario Borreca, Dario D'Antonio, Dario Rosini, Dario Somenzi, Daunia Verdi, David Alessio Merlini, Davide Ferrari, Davide Mascali, Diletta Corallino, Domenico Magagnano, Domenico Rosario Iusco, Domenico Vita, Dorena Caruso, Edoardo Forcignanò, Edoardo Virgilio, Elena Bonati, Eleonora Guaitoli, Elio Francesco Favale, Elisa Bolzoni, Elisa Galasso, Elvira Adinolfi, Emanuela Stratta, Emanuele Caruso, Emanuele Damiano Luca Urso, Emanuele Doria, Emanuele Pontecorvi, Emilio Paolo Emma, Enrico Luzietti, Enrico Pinotti, Erica Monati, Erika Boriani, Ernesto Tartaglia, Ester Marra, Eugenia Rosso, Ezio Lombardo, Fabio Ambrosini, Fabio Carbone, Fabio Crescenti, Fabio Medas, Fabrizio D'Acapito, Federica Chimenti, Federica De Robertis, Federico Cappellacci, Federico Cozzani, Federico Festa, Federico Lovisetto, Federico Maggi, Federico Mazzotti, Filippo D'Agostino, Francesca Ascari, Francesca Di Candido,

Francesca Foglio, Francesca Laura Nava, Francesca Mazzarulli, Francesca Meoli, Francesca Paola Tropeano, Francesca Pecchini, Francesca Pegoraro, Francesco Bagolini, Francesco Belia, Francesco Bianco, Francesco Caldaralo, Francesco Casti, Francesco Cobellis, Francesco Colli, Francesco Colombo, Francesco Madeddu, Francesco Maria Romano, Francesco Matarazzo, Francesco Menegon Tasselli, Francesco Pata, Francesco Salvetti, Francesco Serra, Gabriele Bislenghi, Gabriele Luciano Petracca, Gabriella Lionetto,Gaia Colletti, Gennaro Mazzarella, Gennaro Perrone, Giacomo Anedda, Giacomo Carganico, Giacomo Fuschillo, Gian Andrea Binda, Gian Luca Baiocchi, Gian Luigi Canu, Gianluca Baronio, Gianluca Cassese, Gianluca Fucci, Gianluca Mascianà, Gianluca Pellino, Gianluca Rizzo, Gianluigi Moretto, Gianmario Edoardo Poto, Gianpiero Cione, Giorgio Dalmonte, Giorgio Lisi, Giorgio Rossi, Giovanna Berardi, Giovanna Di Scanno, Giovanna Pavone, Giovanni Battista Damiani, Giovanni Braccini, Giovanni Cestaro, Giovanni Guglielmo Laracca, Giovanni Spiezio, Giovanni Tomasicchio, Giulia Bonfanti, Giulia Cerino, Giulia Maria Francesca Marini, Giulia Turri, Giuliano Barugola, Giuliano Lantone, Giulio Iacob, Giuseppe Candilio, Giuseppe Curro, Giuseppe Frazzetta, Giuseppe Navarra, Giuseppe Palomba, Giuseppe Sica, Giuseppe Trigiante, Gregorio Di Franco, Gregorio Romeo, Guglielmo Clarizia, Guglielmo Giannotti, Guido Mantovani, Guido Sciaudone, Harmony Impellizzeri, Helen Yu, Iacopo Monaci,Ilaria Clementi, Imerio Angriman, Immacolata Iannone, Irnerio Angelo Muttillo, Isabella Ameli, Isabella Pezzoli, Jacopo Guerrini, Jacopo Mercuri, Jacopo Nicolò Marin, Jozel Hila, Laura Fortuna, Laura Olivieri, Leandro Siragusa, Leonardo Solaini, Letizia Santandrea, Lidia Oddis, Ljevin Boglione, Loredana Grezio, Lorenzo Casali, Lorenzo Epis, Lorenzo Gallitiello, Lorenzo Pagliai, Lorenzo Petagna, Lorenzo Ramaci, Lorenzo Tosi, Lorenzo Vona, Luca Amadio, Luca Cestino, Luca Domenico Bonomo, Luca Fabris, Luca Ferrario, Luca Morelli, Luca Perin, Luca Resca, Luca Scaravilli, Lucio Selvaggi, Ludovica Vacca, Ludovico Carbone, Luigi Boccia, Luigi Cayre, Luigi Conti, Luigi Eduardo Conte, Luigi Marano, Maddalena Maria Bignone,

Manuela Mastronardi, Marci Pellicciaro, Marco Anania, Marco Angrisani, Marco Beggiato, Marco Calussi, Marco Clementi, Marco D'Ambrosio, Marco Giacometti, Marco Livrini, Marco Materazzo, Marco Montorsi, Marco Pericoli Ridolfini, Marco Realis Luc, Margherita Carbonaro, Maria Carmela Giuffrida, Maria Di Salvo, Maria Francesca Chiappetta, Maria Grazia Sibilla, Marianna Capuano, Mariarita Tarallo, Marina Valente, Mario Giuffrida, Mario Pacilli, Mario Sorrentino, Mario Trompetto, Marta Breda, Marta Mozzin, Marta Spalluto, Marzia Franceschilli, Marzia Tripepi, Massimiliano Caccetta, Massimiliano Mistrangelo, Matelda Bencini, Matteo Capuzzo, Matteo Rossini, Mattia Marinelli, Maurizio Rho, Maurizio Romano, Maurizio Roveroni, Mauro Marzano, Mauro Montuori, Mauro Podda, Mauro Pozzo, Mauro Santarelli, Micaela Piccoli, Michela Campanelli, Michele Cricrì, Michele Manara, Michele Manigrasso, Michelle Vilardo, Miriam Biancu, Nicholas Rizzi, Nick Salimian, Nicola Busi, Nicola Cillara, Nicola Di Bartolomeo, Nicola Tartaglia, Nicoletta Sveva Pipitone Federico, Nicolò De Santis, Noemi Laquatra, Noemi Zorzetti, Nunzio Velotti, Olivia Boccia, Oreste Claudio Buonomo, Pamela Milito, Paola Batistotti, Paolina Saullo, Paolo Massucco, Paolo Pizzini, Pasquale Losurdo, Patrizia Rubini, Peter Marinello, Pierantonio Cardinale, Pierluigi Lobascio, Pierpaolo Sileri, Pietro Anoldo, Pietro Fransvea, Pietro Giorgio Calò, Raffaele De Filippi, Raffaele Lombardi, Renato Meccariello, Renato Pietroletti, Riccardo Magarini, Riccardo Marsengo, Riccardo Nascimbeni, Roberta Longhin, Roberta Tutino, Roberto Cammara, Rocco Aversa, Sabino Capuzzolo, Salvatore Bonarrigo, Salvatore Bracchitta, Salvatore Carrabetta, Sara Cecconi, Sara Gobbi, Sara Ingallinella, Sara Marzorati, Sayali Valiyeva, Sebastiano Grassia, Serafino Marino, Sergio Sforza, Silvia Curcio, Silvia Neri, Silvia Puddu, Silvio Caringi, Simona Badalucco, Simona Grande, Simona Pisicchio, Simone Berardi, Simone Bosi, Simone Gargarella, Sofia Esposito, Stefania Angela Piccioni, Stefania Bettoni, Stefano Barbieri, Stefano Rossi, Stefaon Scabini, Teresa Perra, Tommaso Farolfi, Tommaso Guagni, Tommaso Loderer, Tommaso Stecca, Tommaso Violante, Ugo Elmore, Ugo Grossi, Umberto Cocozza, Valentina Rampulla, Valentina Sbacco, Valentina Zucchini, Vania Silvestri, Vincenza Paola Dinuzzi, Vincenzo Adamo, Vincenzo La Vaccara, Vincenzo Papagni, Vincenzo Schiavone, Vittoria Bellato, Zullo Alessia.
