# Supplementary material for: Acute diverticulitis management: evolving trends among Italian surgeons. A survey of the Italian Society of Colorectal Surgery (SICCR)
Source: Updates Surg. 2024 Jul 23;76(5):1745–60. doi: 10.1007/s13304-024-01927-y (PMC11455713; doi:10.1007/s13304-024-01927-y)
Supplement: Supplementary file 2 — Supplementary file2 (DOCX 16 KB) [file 13304_2024_1927_MOESM2_ESM.docx]

**Checklist for Reporting Results of Internet E-Surveys (CHERRIES)**

| ***Checklist Item*** | ***Explanation*** |
| --- | --- |
| **Describe survey design** | This survey aims to explore surgeons’ attitude in diagnosis and management in “borderline" situations, either elective or in emergency, that are still not clearly identified by the currently available guidelines and recommendations. The survey has been administered to surgical residents and certified general surgeons (with various experience in general and colorectal surgery) |
| **IRB approval** | No IRB was needed for this type of survey (no ethical concerns have been discussed) |
| **Informed consent** | Participants read and accept terms and conditions. The disclosure form did not require a signature. In “terms and conditions” the length of time to complete the survey, PI and the committee names with data storage details were included. |
| **Data protection** | A locked database on Google form has been used to protect personal data including mail addresses, age and year of residency |
| **Development and testing** | The survey was developed reviewing the literature and basing on previous published surveys on different topics |
| **Open survey versus closed survey** | This was an open survey |
| **Contact mode** | The distribution of the survey took place through mailing lists, instant message services, and the official social media accounts of the Italian Society of Colorectal Surgery (*Società Italiana di Chirurgia Colorettale,* SICCR) on Facebook, Instagram, and LinkedIn. A reminder was mailed two, four and six weeks after the first mailing |
| **Advertising the survey** | Mailing lists, instant message services, and the official social media accounts of the Italian Society of Colorectal Surgery (*Società Italiana di Chirurgia Colorettale,* SICCR) on Facebook, Instagram, and LinkedIn |
| **Web/E-mail** | A link of the survey was sent to all participants which were interested on the topic |
| **Context** | The survey was sent to the mail list of the *Società Italiana di Chirurgia Colorettale,* SICCR and it was published on facebook, Instagram and Linkedin of the Society |
| **Mandatory/voluntary** | This was a voluntary survey |
| **Incentives** | All participants have been included as Collaborators |
| **Time/Date** | November 2022 and February 2023 |
| **Randomization of items or questionnaires** | N/A |
| **Adaptive questioning** | N/A |
| **Number of Items** | 33 items |
| **Number of screens (pages)** | 2 pages |
| **Completeness check** | Manual completeness checks were performed during the data analysis |
| **Review step** | Back button allows to change answers |
| **Unique site visitor** | N/A |
| **View rate (Ratio of unique survey visitors/unique site visitors)** | N/A |
| **Participation rate (Ratio of unique visitors who agreed to participate/unique first survey page visitors)** | Regretfully participations rate was not evaluated |
| **Completion rate (Ratio of users who finished the survey/users who agreed to participate)** | Completion rate was not evaluated |
| **Cookies used** | Cookies not used |
| **IP check** | IP addresses were not recorded. Duplicates were eliminated when questionnaires were fulfilled by the same person (identified by name and email address) |
| **Log file analysis** | No log file analysis used |
| **Registration** | N/A |
| **Handling of incomplete questionnaires** | Only completed questionnaires were analyzed |
| **Questionnaires submitted with an atypical timestamp** | N/A |
| **Statistical correction** | N/A |
